# SARS-COV-2 Vaccination Response in Non-Domestic Species Housed at the Toronto Zoo

**DOI:** 10.3390/vaccines13101037

**Published:** 2025-10-08

**Authors:** Sara Pagliarani, Jaime Tuling, Phuc H. Pham, Alexander Leacy, Pauline Delnatte, Brandon N. Lillie, Nicholas Masters, Jamie Sookhoo, Shawn Babiuk, Sarah K. Wootton, Leonardo Susta

**Affiliations:** 1 Faculty of Science, Syndey School of Veterinary Medicine, The University of Sydney, 410 Werombi Rd., Camden, NSW 2573, Australia; sara.pagliarani@sydney.edu.au; 2 Department of Pathobiology, Ontario Veterinary College, University of Guelph, 419 Gordon St., Guelph, ON N1G 2W1, Canada; jtuling@uoguelph.ca (J.T.); phpham@uoguelph.ca (P.H.P.); aleacy@uoguelph.ca (A.L.); pdelna01@uoguelph.ca (P.D.); blillie@uoguelph.ca (B.N.L.); kwootton@uoguelph.ca (S.K.W.); 3 Department of Wildlife Science, Toronto Zoo, 361A Old Finch Avenue, Toronto, ON M1B 5K7, Canada; nmasters@torontozoo.ca; 4 Canadian Food Inspection Agency, National Centre for Foreign Animal Disease, 1015 Arlington Street, Winnipeg, MB R3E 3M4, Canada; sookhooj@myumanitoba.ca (J.S.); shawn.babiuk@inspection.gc.ca (S.B.); 5 Department of Immunology, Max Rady College of Medicine, University of Manitoba, 750 McDermot Avenue, Winnipeg, MB R3E 0T5, Canada

**Keywords:** Zoetis, subunit vaccine, zoo, polar bears, surrogate neutralization, plaque-reduction test

## Abstract

**Background**: Due to the wide host range of the severe acute respiratory syndrome coronavirus 2 (SARS-CoV-2), vaccination has been recommended for susceptible species in zoological collections, particularly to protect endangered species. The Zoetis^®^ Experimental Mink Coronavirus Vaccine (Subunit) was temporarily authorized in 2021–2024 for emergency use in North America for this purpose. However, there are limited data regarding its safety or efficacy in non-domestic mammals. The present study was conducted to assess the ability of this vaccine to elicit serum neutralizing titers against SARS-CoV-2 in selected animals from the Toronto Zoo (TZ) vaccinated during 2022. **Methods**: Serum samples were collected from 24 individuals across four families (*Cervidae*, *Felidae*, *Ursidae*, and *Hyaenidae*) and tested using a surrogate virus neutralization test (sVNT) and a plaque-reduction neutralization test (PRNT). **Results:** The results showed that all species developed some neutralizing titers after at least one vaccine dose, except for polar bears, which showed no seroconversion. Felids and hyenas had the highest neutralizing titers, which peaked at 3 and declined between 4 and 6 months after boost. These differences may stem from species-specific immune responses or lack of vaccination protocols tailored to individual species. **Conclusions**: While natural infection with SARS-CoV-2 could not be ruled out in the cohort of this study, insights from our results have the potential to inform future vaccine recommendations for non-domestic species. Furthermore, our study highlighted the value of competitive assays in assessing serological responses across a broad range of exotic species, for which reagents, such as anti-isotype antibodies, are often unavailable.

## 1. Introduction

Since its emergence in 2019, the severe acute respiratory syndrome coronavirus 2 (SARS-CoV-2) has demonstrated the ability to naturally infect various animal species, including felids, mustelids (e.g., minks, ferrets), white-tailed deer, and non-human primates (NHPs), among others [1,2,3]. This broad host range highlighted the importance of developing a veterinary-specific vaccine to mitigate zoonotic risks and reduce morbidity and mortality in susceptible animals, in both production and conservation settings. In 2021, Zoetis^®^ developed an experimental SARS-CoV-2 spike protein (S) subunit vaccine for subcutaneous (SC) administration in farmed mink (*Neogale vison*) [4]. The vaccine has proven immunogenic in mink in field trials, as well as in domestic goats and cats (via SC and intramuscular (IM) routes), eliciting neutralizing antibodies against both the Wuhan and Delta variants [5,6]. To address concerns about SARS-CoV-2 in captive wildlife, several North American zoos, including the Toronto Zoo (TZ; Scarborough, ON, Canada), vaccinated select animals under their care with the Zoetis^®^ Experimental Mink Coronavirus Vaccine (Subunit) under conditional license. Species considered at higher risk were selected based on clinical feasibility criteria, calculated affinity of the receptor-binding domain (RBD) of the S protein with the deduced protein sequence of the angiotensin-converting enzyme 2 (ACE-2) for that species [7], and the limited zoo literature available at the time. While initial results showed favorable safety outcomes and good neutralizing titers, especially in big cats [8], data on vaccine immunogenicity in other non-domestic species remain limited. This study aimed at evaluating the immunogenicity of the Zoetis^®^ Experimental Mink Coronavirus Vaccine (Subunit) in select mammalian species at the TZ.

## 2. Materials and Methods

### 2.1. Animal Vaccination and Inclusion Criteria

Animals were vaccinated with the Zoetis^®^ Experimental Mink Coronavirus Vaccine, Subunit (Canadian Food Inspection Agency (CFIA) permit PRVB# 2022007) at the TZ based on species-specific risk assessment [2,3,7,9,10]. The vaccine was administered (either under chemical or behavioral restraint) as a two-dose regimen (prime and booster doses), approximately 1 month apart, using 1 mL of vaccine delivered IM or SC.

For this study, samples were collected either opportunistically, using either frozen sera previously stored or fresh blood from animals restrained for unrelated reasons, or voluntarily, from individuals trained through operant conditioning to provide blood specifically for this research. Banked serum samples (up to early 2020, i.e., before the inception of the pandemic) were included as negative controls when possible. Samples from NHPs were excluded due to biosecurity restriction at the University of Guelph. Animal procedures were conducted in accordance with the animal utilization protocol AUP #2022-03-03, approved by the TZ Animal Care and Research Committee. All samples were collected by TZ staff and serum obtained using standard protocols and stored at −20 °C until use.

### 2.2. Surrogate Virus Neutralization Test (sVNT)

This test evaluates the ability of serum antibodies to disrupt the interaction of the RBD of the SARS-CoV-2 (Wuhan) strain with the human ACE2 (hACE2) [11,12], and does not depend on the availability of species-specific secondary antibodies. The RBD-hACE2 interaction was measured with the GenScript^®^ SARS-CoV-2 Neutralization Antibody Detection Kit (Genscript^®^, cat. no. L00847-A, Rijswijk, 2288EG, The Netherlands). Briefly, 50 μL of serum samples (1:10 diluted) were mixed with an equal volume of recombinant horseradish peroxidase (HRP)-conjugated RBD, incubated for 30 min at 37 °C, transferred into a well of a 96-well plate coated with the recombinant hACE2 receptor, and incubated for 15 min at 37 °C. After washes, binding to the hACE2 was revealed using a colorimetric substrate (3,3′,5,5′-tetramethylbenzidine) for 15 min at room temperature, before stopping the reaction with 1 M sulfuric acid. The percentage of inhibition was determined by comparison to the negative control (no inhibition) provided with the kit, run on every plate, according to this formula: $Inibition=\left(1-\frac{OD\;value\;of\;Sample}{OD\;value\;of\;Negative\;Control}\right)\times \;100\%$. All test sera were run in duplicate. Inhibition ≥ 30% was regarded as a positive neutralization as suggested by the manufacturer’s instructions [12].

### 2.3. Plaque-Reduction Neutralization Test 70% (PRNT70)

This test was carried out at the National Centre for Foreign Animal Disease NCFAD BSL-3 zoonotic laboratory. PRNT70 was conducted on paired pre-vaccination and post-vaccination sera for each animal, using samples with the lowest and highest VNT inhibition values, respectively, if multiple were available. Neutralization against both the Wuhan (ancestral; hCoV-19/Canada/ON-VIDO-01/2020; Drs. Mubareka and Kozak at Sunnybrook Health Sciences Centre, University of Toronto, and VIDO-Intervac at the University of Saskatchewan) and the Omicron (BQ.1) variants were assessed. Viruses were propagated and assayed for PRNT70 in Vero E6 cells overexpressing the transmembrane serine protease 2 (TMPRSS-2) gene (VE6/TMPRSS2; JCRB Cell Bank cat. no. JCRB1819), as previously described [13]. Briefly, the serum samples were heat inactivated (56 °C for 30 min), and two-fold serially diluted starting from undiluted sample. Serum sample dilutions were incubated with ~50 plaque-forming units (PFUs) of SARS-CoV-2 virus at 37 °C for 1 h, and then virus–serum mixtures were transferred to wells containing over 90% confluent VE6/TMPRSS2 cells in 48-well plates. Following 1 h incubation at 37 °C with 5% CO_2_, wells were overlaid with 500 µL of 2% carboxymethylcellulose (Sigma-Aldrich, cat# C5013-500G, Oakville, ON, L6H6J8, Canada). Plates were then incubated at 37 °C for 72 h, fixed with 10% buffered formalin, and stained with 0.5% crystal violet. The highest serum dilutions resulting in a plaque count reduction > 70% compared with virus-only controls were considered neutralizing.

### 2.4. Data Analysis and Descriptive Statistics

To account for the varying collection times, serum samples were grouped into seven temporal windows based on the time elapsed relative to the prime and booster doses: (timepoint #1; TP1) pre-2019 (before 1 January 2019), (TP2) post-January 1, 2019 but pre-prime vaccination, (TP3) within 2 calendar months post-prime but pre-boost, (TP4) within 1 calendar month post-boost, (TP5) 1–2 calendar months post-boost, (TP6) 2–4 calendar months post-boost, and (TP7) > 5 calendar months post-boost. Data derived from the sVNT were analyzed at both single-animal and species level, the latter including all the data points from each animal of a species. GraphPad Prism v.9.4.1 (GraphPad Software, Boston, MA, 02110, USA) was used for descriptive statistics and graph production.

## 3. Results

### 3.1. Animal Demographics and Serum Samples

A total of 65 serum samples were obtained from 24 animals from the families *Felidae* (10), *Cervidae* (4), *Ursidae* (7), and *Hyaenidae* (3). The details regarding basic demographic data and vaccination histories of these animals are summarized in Table 1 and Supplementary Table S1, respectively. All the prime and booster doses were delivered within 2 months of each other, and no vaccine-related adverse reactions were recorded. By TP7, two caribous (#1 and #2) and the jaguar had been euthanized due to chronic degenerative conditions unrelated to vaccination.

Twelve animals (2 lions, 1 jaguar, 1 moose, 3 cheetahs, 1 tiger, 1 hyena, and 3 polar bears) had at least one pre-vaccination and one post-vaccination sample, with all having at least a post-boost data point (total, 51). Seven animals (2 tigers, 2 polar bears, 1 moose, 1 hyena, and 1 lion) had samples exclusively from TP1 or TP2 (total, 9). Five animals (2 brown bears, 2 caribous, and 1 hyena) had exclusively post-vaccination samples (total, 5); these were tested since pre-vaccination samples were initially believed to be available for these animals. Details regarding serum samples distribution and their collection history are outlined in Supplementary Table S2.

### 3.2. Surrogate Virus Neutralization Test (sVNT)

**Figure 1:**
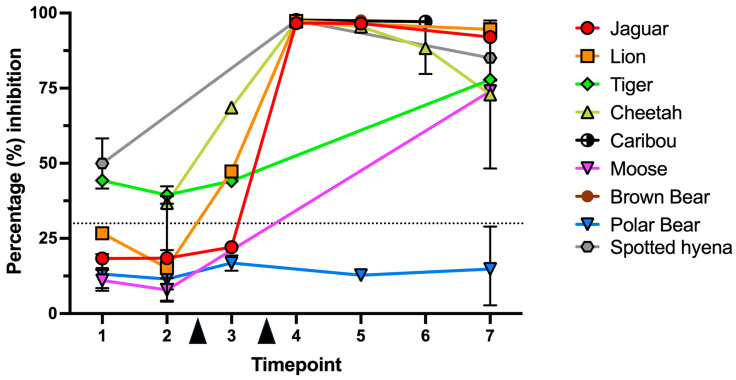
Percentage of surrogate virus neutralization in sera from animals vaccinated at the Toronto Zoo with the Zoetis^®^ Experimental Mink Coronavirus Vaccine (Subunit). All the single-animal data in the cohort are grouped at the species level, including replicates within the same timepoint. Timepoints are defined as time intervals relative to the prime and booster doses (arrowheads). Represented are either single values, or median with range, when multiple values were available for each timepoint. Dotted line is placed at 30% of inhibition, the neutralization threshold according to the Genscript^®^ kit.

Of the 25 samples that were acquired before vaccination (TP1 and TP2), 8 (32%) had a percentage of surrogate neutralization > 30% (threshold). This was observed for 1/3 lions, 2/3 cheetahs, 3/3 tigers, and 2/2 hyenas (Table 2). Surrogate neutralization values, regardless of species, did not differ between TP1 and TP2 (Mann–Whitney test, *p* = 0.1366), suggesting no change in neutralizing titers between pre-2019 samples and those collected between 2019 and before the first vaccine dose. For animals that had both a pre-vaccination and a post-vaccination sample, the relative change in the surrogate neutralization values at the species level was as follows: lion (12.6-fold; *n* = 2), jaguar (5.24; *n* = 1), moose (5.1; *n* = 1), cheetah (5; *n* = 3), tiger (1.97; *n* = 1), hyena (1.86; *n* = 1), and polar bear (1.34; *n* = 3) (Table 2). Regardless of fold-change differences, all these animals had post-vaccination surrogate neutralization values well above 30%, except for polar bears. At TP4-6, caribous and brown bears had surrogate neutralizing values > 90%. While vaccination might have been immunogenic in these species, lack of pre-vaccination samples precluded an appropriate comparison. When collapsing all data points at the species level (Figure 1), all species (except for polar bears) had the highest magnitude of surrogate neutralization (>60%) in the timepoints shortly after the booster dose (TP4-5), with a decline by TP6-7 (i.e., after 4 months).

### 3.3. Plaque-Reduction Neutralization Test 70% (PRNT70)

**Figure 2:**
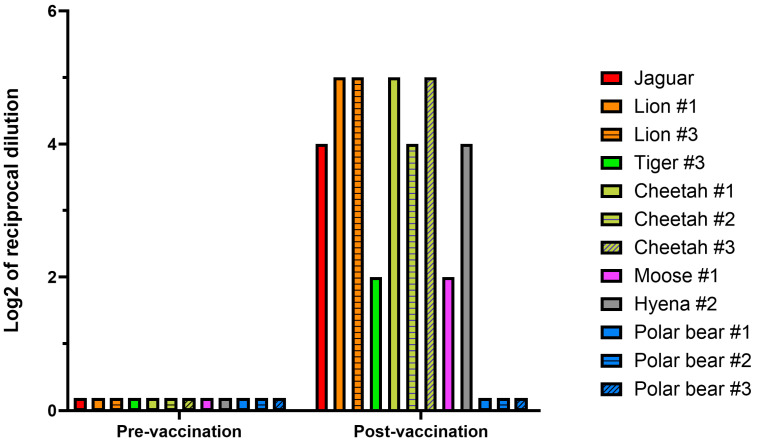
Plaque-reduction neutralization test 70% (PRNT70) against the ancestral SARS-CoV-2 strain, in the sera of animals vaccinated at the Toronto Zoo with the Zoetis^®^ Experimental Mink Coronavirus Vaccine (Subunit). For each animal, paired pre-vaccination and post-vaccination sera were tested. Represented are single values, expressed as log_2_ of reciprocal PRNT70 titer. Lack of neutralizing titer (i.e., lack of neutralization at no serum dilution) is depicted by placeholders on the x axis (i.e., negative).

A PRNT70 assay against the ancestral and Omicron SARS-CoV-2 variants was run on paired pre-vaccination and post-vaccination serum samples from 12 animals (Figure 2). All the post-vaccination samples tested by PRNT70 were collected after the booster dose, except for polar bear #3, which had a post-prime sample tested instead. None of the 12 pre-vaccination samples exhibited neutralizing titers against either virus, regardless of whether they were from TP1 or TP2, even though three of these samples had sVNT values exceeding 30%. All the post-vaccination samples, selected to have the highest sVNT values for that animal, had some degrees of neutralization against the ancestral variant, except for polar bears, which had none. For the jaguar, lions, cheetahs, and hyenas, PRNT70 titers ranged between 1:16 and 1:32, while for the one tiger and the moose it was 1:4. No neutralizing titers were detected against the Omicron variant.

## 4. Discussion

This serological survey showed that different species have varied responses to the Zoetis^®^ Experimental Mink Coronavirus Vaccine (Subunit), although all tested species developed increasing serum neutralizing titers after vaccination, except for the polar bears. Before vaccination, PRNT70 showed no detectable neutralizing activity to SARS-CoV-2 in any animal from this study. After the booster dose, however, most PRNT70 titers against the ancestral strain ranged between 1:4 and 1:32. While certain studies in people suggest that a serum neutralizing titer of 1:16 may be protective [14], there is no clear consensus on serological correlates of protection for SARS-CoV-2 [15]. The sVNT partly mirrored the PRNT70 results, as all the paired samples (except for polar bears) showed an increase in sVNT percentage inhibition after at least one vaccine dose, and all tested species (but polar bears) showed a percentage of sVNT inhibition > 30% after the booster dose. Nonetheless, 8/25 pre-vaccination sample also had >30% inhibition, despite no neutralization being detected by PRNT70 in these samples. The reason for this discrepancy remains unknown. It could be the consequence of cross-reactive antibodies against other coronaviruses (see below), or non-specific reactivity dependent on sample quality (e.g., hemolysis). This underscores the importance of validating sVNT data with other tests, such as neutralization assays. Additionally, it should be noted that sVNT-reactive sera did not show neutralization against the Omicron strain. This was partly expected due to the degree of divergence between these strains, a finding also documented in people [16]

In lions, cheetahs, and the jaguar the vaccine proved immunogenic, showing marked surrogate and PRNT70 neutralizing titers after boost. These findings align with those reported from multiple big cats (*Panthera* spp.) at the San Diego Zoo Safari Park (San Diego, CA, USA) [17] and the AZA (Association of Zoos and Aquariums) Felid Taxon Advisory Group (Felid TAG) [8]. Tigers from our study, however, exhibited relatively lower surrogate and PRNT70 neutralizing titers compared to other felids, and presented with elevated pre-vaccination sVNT titers. This finding may derive from cross-reactive antibodies induced by exposure to other feline coronaviruses (FCoVs), as suspected in another serological survey [18]. Indeed, domestic cats infected with FCoV-1 and FCoV-2 develop cross-reactive antibodies to the RBD of SARS-CoV-2, despite the low overall homology between these viruses (<36%) [19].

The ACE2 of spotted hyenas has a strong calculated binding affinity to the RBD of the SARS-CoV-2 S protein [10], as corroborated by reported cases of natural infection [2]. Our results showed that vaccination with the Zoetis^®^ Experimental Mink Coronavirus Vaccine (Subunit) elicited a strong serum surrogate neutralizing response in two spotted hyenas, which only slightly declined 5 months after boost. Similarly, high sVNT titers following vaccination were observed in serum samples from moose and caribous, with a 5-fold increase in surrogate neutralization activity following vaccination in a moose. Only post-vaccination serum samples were available for the two caribous; however, the very high percentage of surrogate neutralization (>97%) noted for both animals suggests that they had seroconverted.

The absence of sVNT or PRNT70 titers in polar bears may be explained by species-specific immunological differences, serum incompatibility with the in vitro test, or inadequate vaccine protocol, including an insufficient dose or inadequate route of administration. As no peer-reviewed studies are available regarding vaccination in polar bears, contextualization of our results is challenging. The current World Small Animal Veterinary Association guidelines for the vaccination of dogs and cats states that vaccines are not based on a “volume per body mass (size), but rather on the minimum immunizing dose” [20]. Given the larger size of polar bears (median weight of 393 kg (320–475 kg) in this cohort), it is possible to speculate that a higher vaccine dose could have yielded higher serum neutralizing activity. Nonetheless, the second largest mammal in this study showing seroconversion was a 300 kg moose. Lastly, while the vaccine was intended for IM administration, the use of 1–1.5-inch needles by pole syringe likely delivered the vaccine in the subcutaneous fat, which in polar bears can be up to 11.4 cm thick [21]. As a poorly vascularized tissue, the panniculus may have hindered antigen processing, as proposed in human medicine [22].

This study had several limitations, including an opportunistic and limited collection of samples, which resulted in lack of a full set of sera for all the animals included in the study and use of samples collected at different timepoints relative to the prime and booster doses. This sample imbalance also prompted the use of species-level descriptive statistics, which collapsed data from multiple animals of the same species into a single dataset. Nonetheless, single-animal data and fold-change were reported. Similarly, due to the small sample size, statistical analysis comparing magnitude of seroconversion between animals or species was unfeasible.

Lastly, at perhaps more importantly, this study did not account for the possibility of natural SARS-CoV-2 infection, which may have caused seroconversion independently of vaccination. Infection, especially if clinically silent (and therefore not reported in the history) may have contributed to the wide data dispersion observed between animals, even of the same species. While evaluation of serum IgG against SARS-CoV-2 Nucleocapsid protein could have helped distinguish vaccination from infection, lack of species-specific secondary antibodies made development of in-house ELISA test exceedingly complex and was not pursued. Nonetheless, the increase in neutralization titers (both at the taxon level and at a single-animal level when repeated samples were available) after vaccine administration suggests that, at least in part, vaccination played a role in the seroconversion observed in this study’s cohort. Additionally, none of the samples collected at TP2 (i.e., before vaccination but after the inception of the pandemic) showed PRNT70 neutralization titers, suggesting that the ancestral strain might not have been circulating widely at the Zoo at that time. Similarly, none of the serum samples showed neutralizing activity against the Omicron strain. Considering that the vaccination campaign started in the spring of 2022 and that Omicron was present in Canada starting in the fall of 2021 [23], it appears that natural infection with SARS-CoV-2 might not have been common among the animals in our cohort. Lastly, even if natural infection could have been demonstrated, disentangling its contribution from that of vaccination to the overall neutralizing activity of the tested sera remains challenging, which is a limitation inherent to this field study.

## 5. Conclusions

Despite its limitations, this study is among the few to report on the outcomes of an immunization program in a large zoological collection during the COVID-19 pandemic and suggests that most tested taxa were capable of developing serum neutralizing activity following parenteral administration of a SARS-CoV-2 subunit vaccine. Moreover, it underscores the value of competitive assays for assessing serological responses in non-domestic species, where conventional reagents such as species-specific anti-isotype antibodies are often lacking.

## Figures and Tables

**Table 1:** Demographics, SARS-CoV-2 susceptibility assessment, and predicted ACE2–S protein binding affinity of zoo species vaccinated with the Zoetis^®^ Experimental Mink Coronavirus Vaccine (Subunit).

Family	Common Name	Species	Individual Identification	Sex	Age (Y)	Weight (kg)	TZ Risk-Based Assessment *	Calculated Affinity of ACE2 Receptor Binding **
Felidae	Jaguar	*Panthera onca*	Jg1	M	17	76	High risk	Medium
	Lion	*Panthera leo*	Ln1	M	10	200	High risk	Medium
			Ln2	F	10	120	High risk	
			Ln3	F	11	112	High risk	
	Cheetah	*Acinonyx jubatus*	Ch1	M	6	50	High risk	Medium
			Ch2	M	6	51	High risk	
			Ch3	F	10	41	High risk	
	Tiger (Amur, Sumatran)	*Panthera tigris*	Tg1	F	15	82	High risk	Medium
			Tg2	M	14	120	High risk	
			Tg3	M	10	200	High risk	
Cervidae	Moose	*Alces alces*	Mo1	F	9	300	High risk	High
			Mo2	F	9	250	High risk	
	Caribou	*Rangifer tarandus*	Ca1	F	12	105	High risk	High
			Ca2	F	12	112	High risk	
Ursidae	Polar bear	*Ursus maritimus*	Pb1	F	21	365	Medium risk	Low
			Pb2	M	11	465	Medium risk	
			Pb3	M	9	475	Medium risk	
			Pb4	F	7	340	Medium risk	
			Pb5	F	21	320	Medium risk	
	Brown bear	*Ursus arctos*	Bb1	F	23	240	Medium risk	Medium
			Bb2	M	24	550	Medium risk	
Hyenidae	Spotted hyena	*Crocuta crocuta*	Sh1	M	13	50	High risk	High
			Sh2	M	23	70	High risk	
			Sh3	F	10	60	High risk	

* As conducted by the TZ staff based on the literature available at the time [2,3,7,9,10]. ** Indicates the calculated affinity of the SARS-CoV-2 S protein RBD (Wuhan strain) to the deduced amino acid sequence of the ACE2 receptor for each species [10].

**Table 2:** Percentage of surrogate inhibition (>30% threshold) against SARS-CoV-2 in sera sampled at different timepoints (TPs) from select mammals at the Toronto Zoo, after vaccination with the Zoetis^®^ Experimental Mink Coronavirus Vaccine (Subunit).

Species	ID	TP1	TP2	TP3	TP4	TP5	TP6	TP7	Fold Change *
Jaguar	Jg1	18.31	18.45	22.07	96.63	96.50		92.04	5.24
Lion	Ln1		27.11 ** (15.22/39.00)		97.25			92.56	2.50
	Ln2 °	26.71							
	Ln3		4.30	47.30	97.51			96.63	22.70
Cheetah	Ch1		9.90	68.58	97.56	97.17	96.95	48.25	9.85
	Ch2		36.78		96.12	93.78	79.75	97.51	2.65
	Ch3		39.08		97.13				2.49
Tiger	Tg1 °	43.77							
	Tg2 °	44.80							
	Tg3		39.44 ** (42.33/36.55)	44.09				77.87	1.97
Moose	Mo1	14.52	11.76					75.04	5.10
	Mo2 °	7.60	3.99						
Caribou	Cb1 ^†^				97.46				
	Cb2 ^†^				97.91		97.14		
Polar bear	Pb1	16.71		14.27		12.74		14.82	0.89
	Pb2	9.57	8.02					20.75 ** (28.91/12.58)	2.16
	Pb3	19.77	8.98	19.31				8.84 (14.93/2.74)	0.98
	Pb4 °		17.53 ** (21.1/13.96)						
	Pb5 °	8.47							
Brown bear	Bb1 ^†^					97.58			
	Bb2 ^†^				96.95				
Spotted hyena	Sh1 °	53.31							
	Sh2	41.62						77.52	1.86
	Sh3 ^†^				97.51			92.64	

* Fold change, calculated for each animal as the ratio between the highest post-vaccination value (TP3-7) and the highest pre-vaccination value (TP1 or TP2). For animals without pre- or post-vaccination values, the ratio was not calculated. ** For each TP, if more than one value was present, the average is displayed, with single values reported in parenthesis. ° Indicates animals that had only pre-vaccination samples. ^†^ Indicates animals that had only post-vaccination samples.

## Data Availability

All data generated or analyzed during this study are included in this published article and its Supplementary Information files.

## Supplementary Materials

The following supporting information can be downloaded at https://www.mdpi.com/article/10.3390/vaccines13101037/s1; Table S1: Type of restraint, dates of first (prime) and second (booster) doses, and site of administration of the Zoetis^®^ Experimental Mink Coronavirus Vaccine (Subunit) in selected species vaccinated at the Toronto Zoo between March and July 2022; Table S2: Date of serum samples collection, stratified by timepoint (TP), and value of associated surrogate serum neutralization for each animal enrolled in the study, which had been immunized with the Zoetis^®^ Experimental Mink Coronavirus Vaccine (Subunit) at the Toronto Zoo.

## Abbreviations

The following abbreviations are used in this manuscript:

ACE2	Angiotensin converting enzyme 2
CFIA	Canadian Food Inspection Agency
COVID-19	Coronavirus disease 2019
PRNT70	Plaque-reduction neutralization test 70%
RBD	Receptor-binding domain
SARS-CoV-2	Severe acute respiratory syndrome coronavirus 2
sVNT	Surrogate virus neutralization test
TMPRSS-2	Transmembrane serine protease 2
TP	Timepoint
TZ	Toronto Zoo
