# Supplementary material for: SARS-COV-2 Vaccination Response in Non-Domestic Species Housed at the Toronto Zoo
Source: Vaccines (Basel). 2025 Oct 8;13(10):1037. doi: 10.3390/vaccines13101037 (PMC12568043; doi:10.3390/vaccines13101037)
Supplement: Supplementary file 1 [file vaccines-13-01037-s001.zip › vaccines-3870222-supplementary.pdf]

Supplementary Tables

SARS-COV-2 VACCINATION RESPONSE IN NON-DOMESTIC SPECIES HOUSED AT THE TORONTO ZOO

Sara Pagliarani[1], Jaime Tuling[2], Phuc H. Pham[2], Alexander Leacy[2], Pauline Delnatte[2], Brandon N. Lillie[2], Nicholas Masters[3], Jamie Sookhoo[4,5], Shawn Babiuk[4,5], Sarah K. Wootton[2], Leonardo Susta[2*]

Supplementary Table 1. Type of restraint, dates of first (prime) and second (booster) dose, and site of administration of the Zoetis® Experimental Mink Coronavirus Vaccine, Subunit, in selected species vaccinated at the Toronto Zoo between March and July 2022.

Species	Individual identification	Type of restraint^	Prime date of administration	Prime site and route of administration	Booster (days since prime)	Booster site and route administration
Jaguar	Jg1	B / B	2022-03-31	Right thigh, IM	21	Right thigh, SC
Lion	Ln1	D / D	2022-04-30	Right thigh, IM	22	Right thigh, IM
	Ln2	B / B	2022-03-29	Right thigh, IM	23	Right thigh, IM
	Ln3	D /D	2022-04-28	Right thigh, IM	24	Right thigh, IM
Cheetah	Ch1	B / B	2022-04-12	Left thigh, IM	22	Left thigh, IM
	Ch2	B / B	2022-04-12	Left thigh, IM	22	Left thigh, IM
	Ch3	B / B	2022-03-28	Right thigh, SC	25	Right thigh, IM
Tiger (Amur, Sumatran)	Tg1	D /D	2022-04-21	Left thigh, SC	27	Left thigh, SC
	Tg2	D / D	2022-05-18	Right thigh, IM	22	Right thigh, IM
	Tg3	D / D	2022-04-20	Right thigh, IM	62	Right thigh, IM
Moose	Mo1	C / D	2022-03-31	Left gluteal muscle, SC	28	Right thigh, IM
	Mo2	C / D	2022-03-31	Left gluteal muscle, SC	27	Right thigh, IM
Caribou	Ca1*	D/ C	2022-04-10	Left thigh, IM	29	Left thigh, IM
	Ca2*	D / C	2022-04-10	Left thigh, IM	47	Left thigh, IM
Polar bear	Pb1	B / B	2022-04-26	Right thigh, IM	30	Right thigh, IM
	Pb2	B / B	2022-04-26	Right thigh, IM	44	Right thigh, IM
	Pb3	B / B	2022-04-26	Right thigh, IM	30	Right thigh, IM
	Pb4	B / B	2022-04-26	Right thigh, IM	37	Right thigh, IM
	Pb5	B / B	2022-05-11	Right thigh, IM	28	Right thigh, IM
Brown bear	Bb1	D / D	2022-04-13	Right thigh, IM	27	Right shoulder, IM
	Bb2	D / D	2022-04-13	Right thigh, IM	27	Right thigh, IM
Spotted hyena	Sh1	D /D	2022-03-29	Right thigh, IM	23	Right thigh, IM
	Sh2	D / D	2022-04-28	Left thigh, IM	24	Left thigh, IM
	Sh3	D /D	2022-03-29	Left shoulder, IM	23	Left shoulder, IM

SC: Subcutaneous

IM: intramuscular

C: Chemical

B: Behavioural (operant conditioning)

D: Darted 1 mL of vaccine IM without chemical anesthesia

^Type of restraint is indicated as “first dose”/ “second dose’’

*Two animals in this study (Ca1 and Ca2) inadvertently received a third dose of the vaccine on May 27th. 2024. This additional dose was not included in the data presented in the table.

Supplementary Table 2. Date of serum samples collection, stratified by timepoint (TP), and value of associated surrogate serum neutralization for each animal enrolled in the study, which had been immunized with the Zoetis® Experimental Mink Coronavirus Vaccine (Subunit) at the Toronto Zoo.

ID	TP1	TP2	PRIME	TP3	BOOST	TP4	TP5	TP6	TP7
Jg1	-1,483 d^ (18.31)*	-18 d (18.45)	2022-03-31	14 d (22.07)	21 d	15 d (96.63)	50 d (96.50)		228 d (92.04)
Ln1		-810 d (39) -2 d (15.22)	2022-04-30		22 d	10 d (97.25)			185 d (92.56)
Ln2°	- 1,595 d (26.71)		2022-03-29		23 d				
Ln3		-2 d (4.30)	2022-04-28	20 d (47.30)	24 d	24 d (97.51)			185 d (96.63)
Ch1		0 d (9.9)	2022-04-12	22 d (68.58)	22 d	28 d (97.56)	50 d (97.17)	85 d (96.95)	260 d (48.25)
Ch2		0 d (36.78)	2022-04-12		22 d	28 d (96.12)	50 d (93.78)	85 d (79.75)	260 d (97.51)
Ch3		0 d (39.08)	2022-03-28		25 d	14 d (97.13)			
Tg1°	-1,343 d (43.77)		2022-04-21		27 d				
Tg2°	-1,307 (44.80)		2022-05-18		22 d				
Tg3		-506 d (42.33); 0 d (36.55)	2022-04-20	49 d (44.09)	62 d				141 d (77.87)
Mo1	-1,400 d (14.52)	0 d (11.76)	2022-03-31		28 d				238 d (75.04)
Mo2°	-1,400 d (7.60)	0 d (3.99)	2022-03-31		27 d				

Ca1˙			2022-04-10		23 d	24 d (97.46)			
Ca2˙			2022-04-10		23 d	24 d (97.91)		83 d (97.14)	
Pb1	-1,517 d (16.71)		2022-04-26	29 d (14.27)	30 d		46 d (12.74)		314 d (14.82)
Pb2	-1,395 (9.57)	-7 d (8.02)	2022-04-26		44 d				161 d (28.91); 339 d (12.58)
Pb3	-1,486 d (19.77)	-7 d (8.98)	2022-04-26	29 d (19.31)	30 d				175 d (14.93) 343 d (2.74)
Pb4°		-7 d (21.1) -6 d (13.96)	2022-04-26		37 d				
Pb5°	-1,410 (8.47)		2022-05-11		28 d				
Bb1˙			2022-04-13		27 d		34 d (97.58)		
Bb2˙			2022-04-13		27 d	26 d (96.95)			
Sh1°	-1,516 d (53.31)		2022-03-29		23 d				
Sh2	-1,374 d (41.62)		2022-04-28		24 d				366 d (77.52)
Sh3˙			2022-03-29		23 d	12 d (97.51)			376 d (92.64)

Timepoint #1 (TP1): Samples collected before January 1st, 2019.

TP2: Samples collected after January 1st, 2019, and before prime date.

TP3: Samples collected up to 2 calendar months after prime date.

TP4: Samples collected up to 1 calendar month after booster vaccination.

TP5: Samples collected between 1 and 2 calendar months after booster vaccination.

TP6: Samples collected between 2 to 4 calendar months after booster vaccination.

TP7: Samples collected after November 1st, 2022 (more than 5 calendar months after boost vaccination).

^Indicates the interval (in days) between sample collection and prime date (TP1, TP2, TP3), prime and booster dates (BOOST), and between sample collection and booster date (TP4-7). For TP1 and 2, values are negative to indicate days before prime. A value of 0 days indicates that the sample was collected at the time of prime or booster dose.

°Indicates animals that had only pre-vaccination samples

˙Indicates animals that had only post-vaccination samples

*The value in parenthesis by the date indicates the percentage of surrogate virus neutralization for the samples collected on that day. If one time point contains more than one sample, two values in parenthesis are provided.
